# Development of myelinating glia: An overview

**DOI:** 10.1002/glia.24238

**Published:** 2022-07-04

**Authors:** Carlo D. Cristobal, Hyun Kyoung Lee

**Affiliations:** ^1^ Integrative Program in Molecular and Biomedical Sciences Baylor College of Medicine Houston Texas USA; ^2^ Jan and Dan Duncan Neurological Research Institute Texas Children's Hospital Houston Texas USA; ^3^ Department of Pediatrics Baylor College of Medicine Houston Texas USA; ^4^ Department of Neuroscience Baylor College of Medicine Houston Texas USA

**Keywords:** CNS and PNS development, myelination, oligodendrocytes, Remak cells, Schwann cells

## Abstract

Myelin is essential to nervous system function, playing roles in saltatory conduction and trophic support. Oligodendrocytes (OLs) and Schwann cells (SCs) form myelin in the central and peripheral nervous systems respectively and follow different developmental paths. OLs are neural stem‐cell derived and follow an intrinsic developmental program resulting in a largely irreversible differentiation state. During embryonic development, OL precursor cells (OPCs) are produced in distinct waves originating from different locations in the central nervous system, with a subset developing into myelinating OLs. OPCs remain evenly distributed throughout life, providing a population of responsive, multifunctional cells with the capacity to remyelinate after injury. SCs derive from the neural crest, are highly dependent on extrinsic signals, and have plastic differentiation states. SC precursors (SCPs) are produced in early embryonic nerve structures and differentiate into multipotent immature SCs (iSCs), which initiate radial sorting and differentiate into myelinating and non‐myelinating SCs. Differentiated SCs retain the capacity to radically change phenotypes in response to external signals, including becoming repair SCs, which drive peripheral regeneration. While several transcription factors and myelin components are common between OLs and SCs, their differentiation mechanisms are highly distinct, owing to their unique lineages and their respective environments. In addition, both OLs and SCs respond to neuronal activity and regulate nervous system output in reciprocal manners, possibly through different pathways. Here, we outline their basic developmental programs, mechanisms regulating their differentiation, and recent advances in the field.

## Main Points

- Oligodendrocytes (OLs) and Schwann cells (SCs) share some characteristics including transcription factors, myelin components, and responsiveness to neuronal activity
- OLs and SCs use unique mechanisms to form myelin and possess differential capacities to respond to external factors
- The lineage and environment of OLs and SCs may account for differences in their developmental plasticity and regenerative capacity.

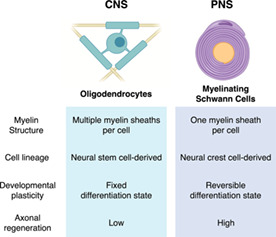

Myelin is an essential component of the nervous system. Myelin has been canonically known to facilitate saltatory conduction in axonal fibers and speed up electrical conduction (Hartline & Colman, 2007; Salzer et al., 2016; Zalc, 2016). More recent discoveries have uncovered many other roles that myelin sheaths play, including metabolic support of axons and modulation of neuronal activity and higher nervous system functions (Herbert & Monk, 2017; Jessen & Mirsky, 2019a, 2019b; McKenzie et al., 2014; Simons & Nave, 2016). As such, defects or loss of myelin through disease or injury can result in a variety of neurological phenotypes including cognitive and motor deficits (Gibson et al., 2018; Kamil et al., 2019). Myelin sheaths in the central nervous system (CNS) and peripheral nervous system (PNS) are produced by distinct myelinating cells: oligodendrocytes (OLs) in the CNS and Schwann cells (SCs) in the PNS. OLs and SCs have major differences in structure, function, and development that make them uniquely suited to each system's needs.

In this review, we aim to answer key questions in CNS and PNS myelination: (1) When and where do OLs and SCs originate from? (2) What are the key pathways that regulate CNS and PNS myelination? and (3) What are recent advances in our understanding of CNS and PNS myelinating glia? Attempting to answer these questions is crucial to uncovering differences in their regenerative capacity and finding novel therapeutic strategies that can stimulate myelination in each or both systems.

## 1 ORIGIN AND TIMING OF OLIGODENDROCYTE DEVELOPMENT

**FIGURE 1:**
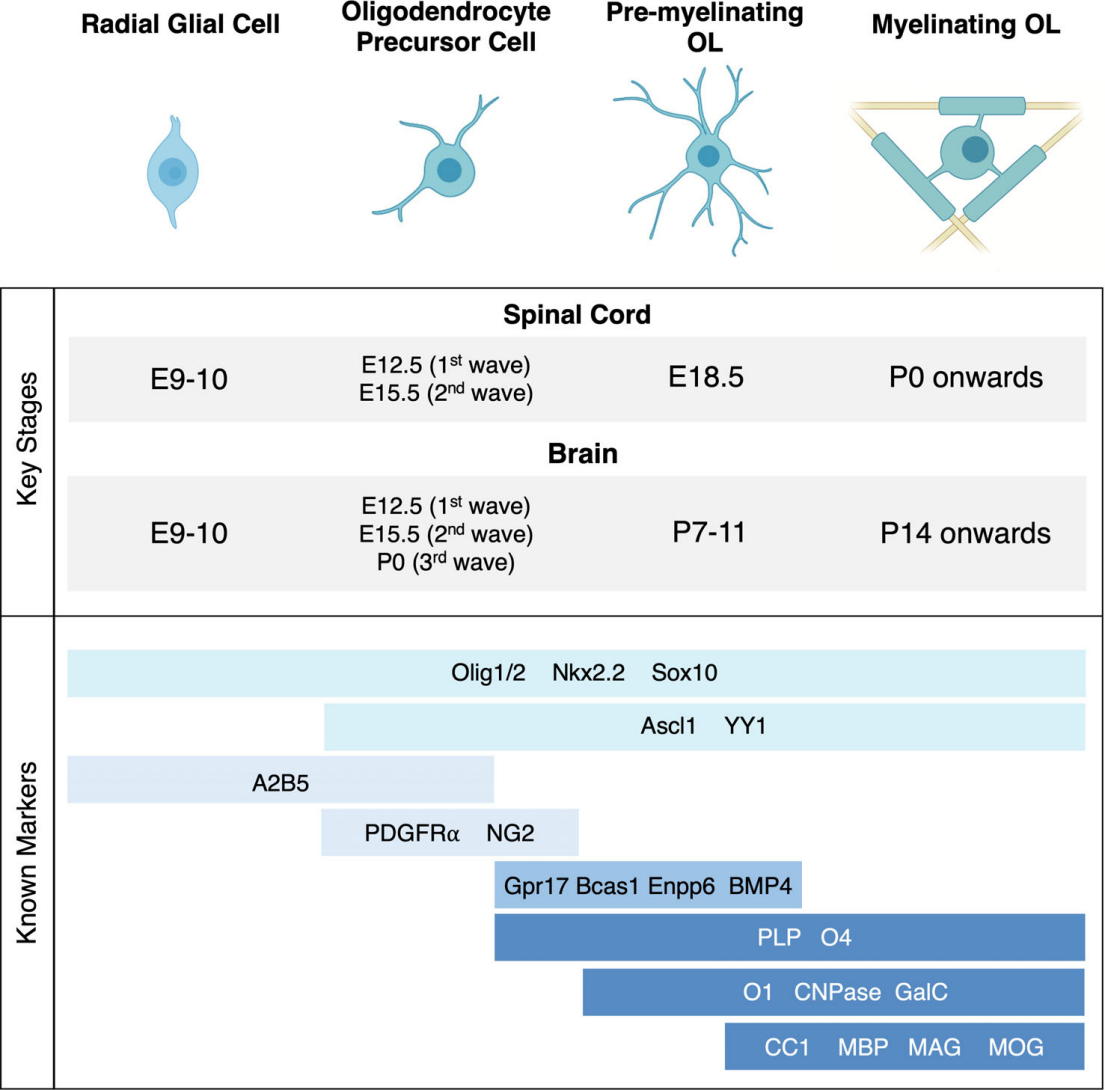
Oligodendrocyte (OL) development and associated markers. (Top) after specification from radial glial cells during early embryonic development, OL lineage cells undergo 3 distinct stages of differentiation. In mice, OL precursor cells (OPCs) are generated in 2 major waves in the spinal cord and in 3 major waves in the brain. The pool of OL‐lineage cells develops earlier in the spinal cord, which undergoes active myelination as early as birth. On the other hand, in the brain, OL maturation and myelination begin to peak at 2–4 weeks after birth. Myelination continues throughout adulthood in both regions. (Bottom) each stage of OL development is defined by key molecular markers. A subset of markers is expressed from radial glial cells all the way to myelinating OLs, and in all stages of the OL lineage after specification. New sequencing technologies have enabled the discovery of more stage‐specific markers, adding to this growing list.

Most OLs are originated through the following basic differentiation program: (1) after neurogenesis, neural stem cell‐derived radial glia become specified as OL precursor cells (OPCs); (2) OPCs proliferate and migrate across the CNS at specific time points known as ‘waves’; (3) a subpopulation of OPCs undergo partial differentiation into pre‐myelinating OLs while many remain OPCs until adulthood; and (4) pre‐myelinating OLs form compact myelin sheaths and become mature myelinating OLs. Each of these stages is defined by a growing set of cellular markers, as shown in Figure 1.

OPC specification and production occurs at different time points and locations across the CNS. In the mouse spinal cord, the first wave of OPCs originate from the ventricular germinal zones at E12.5. These OPCs are derived from the ventral pMN progenitor domain that also generates motor neurons (Pringle & Richardson, 1993; Timsit et al., 1995). After motor neurons are produced, pMN progenitors give rise to ventrally‐derived OPCs that proliferate and migrate rapidly in all directions, becoming evenly distributed throughout the spinal cord by E15 (Hajihosseini et al., 1996; Orentas & Miller, 1996; Pringle & Richardson, 1993). Soon after, a second wave of OPCs is produced from the dorsal part of the spinal cord at E15.5. These OPCs differentiate from a small subset of radial glia, and eventually proliferate and migrate throughout the spinal cord as well (Cai et al., 2005; Fogarty et al., 2005). Ventrally‐derived OPCs make up an estimated 80% of all OPCs in the mouse spinal cord, with the dorsally derived OPCs comprising the remaining 20% (Bergles & Richardson, 2015; Fogarty et al., 2005; Tripathi et al., 2011).

In the brain, OPCs are also produced in three distinct competitive waves, as first demonstrated in seminal lineage tracing studies in mice (Kessaris et al., 2005). At E12.5, OPCs appear in the ventral ventricular zone (VZ) of the medial ganglionic eminence (MGE), constituting the first wave, and are soon after produced in a second wave from the lateral ganglionic eminence (LGE) at E15.5. These OPCs begin to migrate dorsally and laterally from ~E16 in the mouse brain (Kessaris et al., 2005; Richardson et al., 2006; Tripathi et al., 2011). The third wave of OPCs is produced after birth, starting from the cortical VZ and migrating out into the rest of the brain. Interestingly, the first wave of MGE‐derived OPCs are eventually eliminated from the cortex and the second and third waves of OPCs predominate the OPC population in the brain (Kessaris et al., 2005; Tripathi et al., 2011). In the cortex for instance, cortical VZ‐derived OPCs from the third wave are estimated to comprise about 80% of the population, with the remaining 20% coming from the LGE‐derived second wave (Bergles & Richardson, 2015; Tripathi et al., 2011).

OPCs remain evenly distributed throughout the CNS throughout life in both gray and white matter (Chang et al., 2000; Dawson et al., 2003; Pringle et al., 1992). While they comprise a reserve population of cells ready to proliferate, migrate, and differentiate into OLs, they are also thought to serve a variety of functions in the CNS (Bergles & Richardson, 2015; Rivers et al., 2008). Neurons have been found to form active synapses with OPCs (Bergles et al., 2000; Bergles & Richardson, 2015), and emerging evidence in mice and zebrafish has shown that OPCs may play important roles in phagocytosis, immune‐like functions, synapse‐mediated regulation of neuronal activity, and axonal remodeling during circuit formation in the CNS (Benamer et al., 2020; Buchanan et al., 2021; Xiao et al., 2022). Recent single‐cell RNA sequencing in mice combined with functional and biochemical studies revealed key differences in OPCs in the brain and spinal cord during development. Interestingly, spinal cord OPCs were found to have higher levels of cholesterol biosynthesis and uptake enzymes and differential dependence on mTOR signaling (Khandker et al., 2022). This reveals region‐specific and functional heterogeneity in the OPC population, but the underlying mechanisms leading to these differences remain to be uncovered.

A subset of OPCs proceed through the developmental program to form pre‐myelinating OLs. Pre‐myelinating OLs are defined by having highly branched morphologies and expressing the myelin protein PLP. In rodents, the PLP promoter is transiently active as OPCs delaminate from the VZ at E12.5, and single‐cell RNA sequencing experiments confirm low levels of PLP mRNA in the OPC stages (Harlow et al., 2014; Marques et al., 2016; Timsit et al., 1995). Levels of PLP protein are then upregulated to high levels beginning in pre‐myelinating OLs during postnatal development (Marques et al., 2016; Trapp et al., 1997). More recently, protein and mRNA levels of the myelin associated protein BCAS1 were found to increase in pre‐myelinating OLs in human and mouse tissues (Fard et al., 2017), and transcripts of BMP4 and ENPP6 in mice, potentially serving as new distinct markers for this intermediate stage (Hughes & Stockton, 2021; Marques et al., 2016; Xiao et al., 2016; Zhang et al., 2021). Pre‐myelinating OLs eventually undergo phenotypic and transcriptional changes and form compact myelin sheaths as they differentiate into fully mature, myelinating OLs. In the mouse spinal cord, some OPCs begin to express pre‐myelinating markers at E18.5, while in the brain, pre‐myelinating OLs can be observed after birth and throughout adulthood (Rivers et al., 2008). Longitudinal imaging studies in the mouse cortex have demonstrated that OL maturation is highly inefficient, with most cells not differentiating beyond the pre‐myelinating stage. From early development until adulthood in the mouse, OPCs continuously exit the cell cycle and generate pre‐myelinating OLs, but only ~22% of pre‐myelinating OLs fully differentiate into myelinating OLs. The majority of pre‐myelinating OLs instead undergo apoptosis if they fail to myelinate after a period of about 2 days (Hughes et al., 2018). Thus, the pre‐myelinating stage has been identified as a bottleneck in OL development, and shifting the decision from apoptosis to differentiation remains an active area of investigation to promote myelination (Hughes et al., 2018; Hughes & Stockton, 2021).

As they initiate myelination, OL lineage cells begin to express CNPase and GalC proteins. While low levels of their transcripts may be found as early as the OPC stage in mice, protein levels are upregulated significantly early in myelin formation. As they continue to differentiate, myelin‐forming cells begin to express higher protein levels of the mature marker APC/CC1 and the myelin protein MBP; these are transcribed and synthesized at various subcellular locations and transported to the growing myelin sheath. For instance, MBP mRNA is transported in cytoplasmic granules and synthesized at the distal process (Simons & Nave, 2016; Snaidero et al., 2014; Wake et al., 2011). These myelin proteins are turned over throughout life and continuously synthesized to maintain functional sheaths (Meschkat et al., 2022). Myelin formation also requires the synthesis of a large amount of lipids, which is estimated to comprise up to 80% of its dry weight. Although no lipid species are unique to the sheath structure, myelin is comprised of a particular mix of lipids, with an approximate 2:2:1 ratio for cholesterol, various phospholipids, and glycolipids derived from both endogenous and dietary sources (reviewed in Schmitt et al., 2015 and Montani, 2021). Once compact myelin is formed, cytosolic channels remain as part of the sheath, providing a cytosolic compartment bridging the OL and axon. These interfaces contain ion channels, glutamate receptors, and transporters that play crucial roles in metabolic support of axons (Snaidero et al., 2014, 2017).

In the mouse brain, myelination peaks between 2–4 weeks after birth, and continues for at least 8 postnatal months at a decreasing rate (Rivers et al., 2008). Using longitudinal two‐photon imaging, recent work has shown that myelin sheaths are extremely stable and that oligodendrogenesis and myelination are dynamic processes, with over half of the final amount of OLs in the mouse somatosensory cortex being generated after 4 months of age (Hughes et al., 2018). Differences in sheath thickness and length between brain and spinal cord OLs in the mouse CNS have been previously established (Bechler et al., 2015; Khandker et al., 2022). In addition, labeling studies using stage‐specific fluorescent‐tagged mice have demonstrated major differences in the survival of myelinating OLs between the brain and spinal cord throughout life. While ~90% of myelinating OLs present at P60 remained 18 months later in the corpus callosum, less than 60% were present in the spinal cord during the same period (Tripathi et al., 2017). The difficulty of performing longitudinal two‐photon imaging in the spinal cord has precluded detailed studies on sheath formation and oligodendrogenesis, but the combined evidence points to region‐specific differences in myelination and survival. These differences can be explained in part due to region‐specific changes in developmental pathways; for instance, the loss of mTOR/Raptor and NRG1 signaling axes have differential impacts on OL development between different regions of the mouse CNS (Bercury et al., 2014; Taveggia et al., 2008; Wahl et al., 2014). However, it is unclear how much is due to differences in environment or to cell‐intrinsic properties of OLs in each region, and further studies will be required to understand these aspects of development. Other advances in myelin plasticity and function will be highlighted later in this review.

## 2 ORIGIN AND TIMING OF SCHWANN CELL DEVELOPMENT

**FIGURE 2:**
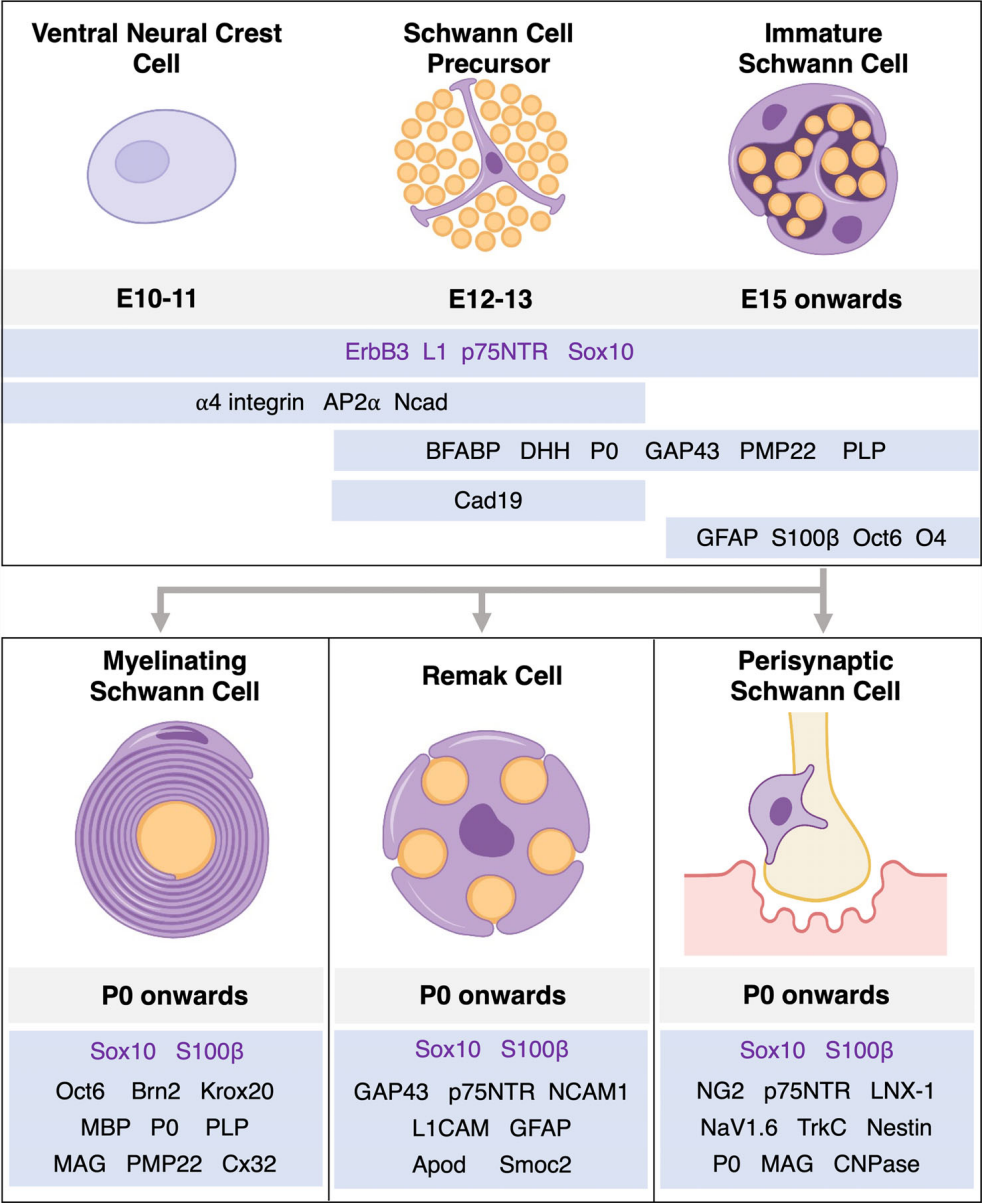
Schwann cell (SC) development and associated markers. (Top) Early stages of SC development occur during embryonic stages in mice. Neural crest cells develop into mature SCs only after undergoing two well‐defined intermediate stages. SC precursors (SCPs) are intimately interspersed between axons, relying on axon‐derived signaling to survive. Immature SCs (iSCs) begin to express differentiation markers, rely on autocrine signaling for survival, and facilitate the process of radial sorting. (Bottom) axon‐associated signals induce iSCs to differentiate into myelinating and non‐myelinating Schwann cells during perinatal and postnatal stages. Each of these differentiation states are thought to be plastic and depend on axon‐derived and environmental cues for their maintenance. While expressing distinct combinations of molecules, each of these fates are known to express pan‐SC lineage markers Sox10 and S100β.

Schwann cells (SCs) originate from neural crest cells (NCCs) and have unique and flexible developmental pathways that allow for distinct, reversible differentiation outcomes. The basic developmental program for axon associated SCs is as follows: (1) NCCs specify into Schwann cell precursors (SCPs); (2) SCPs develop into immature SCs (iSCs), which can then differentiate into at least four potential cellular fates; (3) a subset of iSCs differentiate into myelinating SCs which wrap the axons, and another subset differentiates into non‐myelinating SCs, including but not limited to Remak SCs and perisynaptic SCs (see Figure 2). Other cell SCP‐derived fates also exist and will be briefly covered in this review.

NCCs are a lineage of cells that give rise to a variety of cell types, including melanocytes, skeletal and connective tissue in the head, cardiac cells, and PNS cells (Monk et al., 2015; Woodhoo & Sommer, 2008). NCCs originate from the dorsal‐most region of the developing neural tube, and cells originating from the trunk region give rise to most neural crest‐derived cells in the PNS (Theveneau & Mayor, 2012). Starting from E10‐11 in mice, trunk NCCs migrate ventrally and dorsolaterally (Jessen & Mirsky, 2005), and of these, ventrally‐migrating NCCs give rise to many PNS cells including sensory DRG neurons, sympathetic neurons, and SCPs (Le Douarin & Teillet, 1974; Weston, 1963). In addition to trunk NCCs, a subpopulation of neural crest‐derived cells called boundary cap cells also give rise to SCs of the dorsal and ventral roots (Marol et al., 2004).

At E12‐13 in mice, SCPs begin to appear in early embryonic nerve structures. At this stage, axons are organized into tightly packed structures, which have glial cell processes and cell bodies interspersed between them. These structures lack any significant extracellular matrix or basal lamina and are almost exclusively axons and SCPs (Jessen & Mirsky, 2005). SCPs are known to be migratory, proliferative, and acutely dependent on axon‐associated survival signals (Dong et al., 1995; Monk et al., 2015). In a reciprocal manner, SCPs are also implicated in the survival and terminal targeting of the neurons they interact with. Axons grown without SCPs have been found to follow a normal position and trajectory but have abnormal branching and synapse formation. Indeed, SCPs form complex scaffolds at axon terminals and are thought to be key players in synapse formation during PNS development (Birchmeier, 2009; Wanner et al., 2006). Many myelin‐associated proteins such as PLP, PMP22, and P0 have been found to be present at the SCP stage in embryonic mouse development. The PLP promoter is active at the SCP stage in embryonic mice (Griffiths et al., 1998), and other myelin‐associated genes such as PMP22, P0, and FABP7 as well as various ECM components and receptors were found to be present at this stage at both the transcript and protein level, albeit at lower levels compared to later stages (Buchstaller et al., 2004; Haack & Hynes, 2001; Hagedorn et al., 1999; Jessen & Mirsky, 2019b). Importantly, the cell surface molecule Cad19 is the only gene currently known to be upregulated selectively at the SCP stage and not in other stages of SC development (Jessen & Mirsky, 2019b; Takahashi & Osumi, 2005).

At E13‐15 in mice, SCPs differentiate into iSCs, as evidenced by the expression of key markers including GFAP, O4, and S100β at both the transcript and protein levels (Buchstaller et al., 2004; Jessen et al., 1990; Jessen & Mirsky, 2005). Unlike SCPs, iSCs cease to be migratory and become dependent on autocrine factors for survival instead of axon‐derived factors (Meier et al., 1999). They begin to deposit ECM proteins organized into a nascent basal lamina structure as they envelop groups of axons organized in columns or families. During these stages, iSCs serve two important functions: (1) they initiate the process of radial sorting, which separates large‐caliber axons destined to become myelinated from those that will remain in groups called Remak bundles; and (2) they promote the differentiation of other cellular components of the PNS through multiple signaling pathways (Jessen & Mirsky, 2005; Mukouyama et al., 2005; Parmantier et al., 1999; Webster et al., 1973).

The process of radial sorting begins perinatally at E19‐20 in mice and continues until about P10 (Feltri et al., 2016; Monk et al., 2015). In this process, large‐caliber axons segregate out of their families to become ensheathed by an iSC individually (Webster et al., 1973). This occurs as SCs match axon numbers through dynamic proliferation and apoptosis. A brief overview of radial sorting is as follows (reviewed in Jessen & Mirsky, 2005 and Feltri et al., 2016): in early stages of radial sorting, iSCs deposit ECM components, organizing them into an immature basal lamina. These iSCs then infiltrate within the nerve structure, subdividing axons into smaller bundles that are surrounded by multiple iSCs. These surrounding iSCs then send lamellipodia‐like processes into the bundles, separating larger caliber axons into the periphery. Once the larger axons are in place, a process called defasciculation begins. Here, an iSC divides, producing a daughter cell that completely separates one large axon from the bundle and creates its own basal lamina around it. The daughter cell then differentiates into a promyelinating SC and can begin the process of myelination. After all large caliber axons are separated for myelination through this process, iSCs associated with bundles of smaller‐caliber axons can then differentiate into Remak SCs which form their own mature basal lamina (Feltri et al., 2016; Jessen & Mirsky, 2005; Webster et al., 1973).

Parallel to radial sorting, iSCs also play a major role in the maturation of other cellular components of the nerve. Signals from iSCs are known to be crucial for the formation of nerve‐associated arteries as well as the perineural and epineural sheaths that surround the nerve; for instance, iSCs secrete VEGF, stimulating mesenchymal cells to differentiate into arteries (Mukouyama et al., 2005), as well as Desert hedgehog (Dhh) for the formation of the perineurium and epineurium (Parmantier et al., 1999). In addition, iSCs also play an important role in injury response, communicating with fibroblasts to promote basal lamina formation which then facilitates reinnervation and remyelination (Parrinello et al., 2010).

At about the time of birth, iSCs can begin to differentiate into either myelinating or non‐myelinating SCs after radial sorting (Woodhoo & Sommer, 2008). SCs that surround individual large‐caliber axons become promyelinating SCs, wrapping the axon around 1–1.5 times, and begin to express transcription factors that induce the expression of proteins involved in cellular signaling, myelin structure, and lipid biosynthesis (Herbert & Monk, 2017; Monk et al., 2015). These include proteins in compact myelin (P0, MBP, PMP22), noncompact myelin (Connexin‐32, MAG), and enzymes for production of cholesterol, cerebroside, and sphingomyelin (Monk et al., 2015). As these differentiation programs proceed, SCs begin to fully wrap their individual associated axons, beginning from the cytoplasmic inner tongue to eventually form compact myelin (Salzer, 2015).

As myelination occurs, other iSCs also differentiate into nonmyelinating SCs, which include Remak SCs and perisynaptic SCs (Griffin & Thompson, 2008). Remak SCs are associated with smaller caliber axons that are organized into single bundles, with the number of axons differing depending on their location across the nerve. As iSCs mature into Remak SCs, they continue to produce ECM components around the bundle, forming a mature basal lamina (Griffin & Thompson, 2008). Remak SCs play important roles in trophic and metabolic support of axons, although the exact mechanisms remain unclear (Beirowski et al., 2014; Bouçanova & Chrast, 2020; Viader et al., 2011). Remak SCs share the expression of many protein markers with iSCs such as NCAM1 and GFAP (Jessen et al., 1990; Mirsky et al., 2008; Triolo et al., 2006), and recent work has revealed more specific markers such as Apod and Smoc2 at the transcript level (Wolbert et al., 2020). Apart from Remak SCs, iSCs also differentiate into perisynaptic SCs, which associate with axons at neuromuscular junctions (NMJs). These cells form a loose cap over synaptic sites at birth and participate in synapse formation and refinement postnatally (Sanes & Lichtman, 1999; Wolpowitz et al., 2000). Perisynaptic SCs express muscarinic and purinergic receptors (Young et al., 2005; Zuo et al., 2004) and exhibit intracellular Ca^2+^ waves upon receptor activation, although the function of these waves remain unclear (Todd et al., 2007). In addition, they express several myelin proteins (Georgiou & Charlton, 1999) and have been found to be essential for axon guidance and remodeling after injury (Kang et al., 2014).

One unique aspect of SC development is the plasticity of their differentiated phenotypes. In adult nerves, SCs have been found to retain the capacity to alternate between different phenotypes in response to environmental signals, and it is thought that Remak and myelin SC phenotypes are mostly determined by the axons they associate with (Jessen et al., 2015). Both Remak and myelin SCs can give rise to a distinct repair SC (or Bungner SC) phenotype after injury (Jessen & Mirsky, 2008). The robust regenerative potential of the PNS can be greatly attributed to this adaptive nature, which supports repair and reinnervation. To produce the repair phenotype, myelin‐associated genes are downregulated and the molecules that are expressed in the iSC stage are upregulated (Jessen & Mirsky, 2008). In addition, neurotrophic factors and pro‐inflammatory cytokines begin to be produced and a cell‐intrinsic myelin breakdown process is initiated (Rotshenker, 2011; Vidal et al., 2013). Unlike the OL which has a stable differentiated state, this level of plasticity is unique to the SC and allows it to facilitate more effective repair. However, this is also thought to make SCs vulnerable to damage from immune assaults, genetic abnormalities in demyelinating neuropathies, and in tumor formation (Jessen et al., 2015).

## 3 SIGNALING PATHWAYS AND TRANSCRIPTIONAL MECHANISMS REGULATING CNS MYELINATION

### 3.1 Specification and maintenance of the OPC state

In the developing CNS, the specification of OPCs is tightly controlled by diffusible morphogens that determine the expression of downstream intracellular factors. In the developing spinal cord, BMPs and Wnts from the roof plate and ectoderm of the dorsal midline as well as Shh from the floor plate and notochord at the ventral midline determine the establishment of the pMN domain where OPCs are generated, as shown in chick and mouse embryos (Orentas & Miller, 1996; Poncet et al., 1996; Pringle et al., 1996). Notch signaling through the Delta/Jagged family of ligands has also been demonstrated to cooperate with Shh signaling for specification, as first demonstrated in chick and zebrafish spinal cords (Agius et al., 2004; Mekki‐Dauriac et al., 2002; Park & Appel, 2003). Jagged2, a Notch ligand, has been found to inhibit OPC production in mouse and chick embryos until the time that pMN cells switch from generating motor neurons to generating OPCs (Rabadán et al., 2011). As OPCs specify in the pMN domain, Shh activates expression of OL‐lineage genes through its downstream Gli protein mediators. One of these genes is the transcription factor Olig2, which is necessary to define the pMN domain and promotes OL‐lineage gene expression, as first clearly demonstrated in studies using chick and zebrafish systems (Park et al., 2002; Zannino & Appel, 2009; Zhou et al., 2001). Other transcription factors such as Nkx6.1 and Gli2 also influence OPC production less directly by defining the borders of the pMN domain, and OPC specification is reduced in their absence (Liu et al., 2003; Qi et al., 2003).

Olig2 is necessary for generating ventrally derived OPCs as well as motor neurons. This dual role is modulated by phosphorylation of Olig2 at Ser147 which promotes motor neuron specification, whereas, at later developmental timepoint, dephosphorylation at this site shifts the balance to producing OPCs. Olig2^S147A^ transgenic mice, in which Olig2 phosphorylation at S147 cannot occur, lack the pMN precursor domain at E11.5 and fail to develop OPCs at E14.5; however, they produce an OPC population later in development at E18.5, suggesting a stage‐specific role for Olig2. It is thought that Olig2 dephosphorylation causes it to sequester Ngn2, a proneural transcription factor (Li et al., 2011). While Olig2 is vital for ventral OPCs and plays a broad role in promoting the OL lineage fate, it is not the only factor responsible for OPC specification. OPCs can also arise from more dorsal regions of the neural tube that do not express Olig2 or Nkx6 genes at these stages. This may be explained through potential compensation by Olig1, especially in the hindbrain (Cai et al., 2005; Kessaris et al., 2005; Richardson et al., 2006; Vallstedt et al., 2005). While Olig1 is thought to be dispensable for OPC specification, OL development and myelination, it appears to be necessary for remyelination in mice, as Olig1‐null mice develop normal‐appearing myelin but fail to remyelinate (Arnett et al., 2004; Emery & Lu, 2015). Other transcription factors that contribute to OPC specification are Ascl1, Sox9, and NFIA. While these genes are not exclusively expressed in the OL lineage, deletion or knockdown of these factors in mouse and chick models have led to reduction in OPC populations early in development, revealing potential roles in specification (Deneen et al., 2006; Kang et al., 2012; Stolt et al., 2003; Sugimori et al., 2008).

After specification, OPCs migrate and proliferate to spread throughout the CNS. At this stage, they have continued expression of Olig1 and Olig2, which suggest essential roles in the maintenance of the OPC phenotype (Emery & Lu, 2015; Zhou & Anderson, 2002). Olig2 has been found to induce the expression of transcription factors Nkx2.2 and Sox10 in OPCs in the mouse and chick spinal cord (Küspert et al., 2011; Zhou et al., 2001). Nkx2.2 is a pro‐differentiation factor that is not essential for OPC maintenance but has been shown to be important in OL differentiation (Kucenas et al., 2008; Liu et al., 2007; Zhou et al., 2001). On the other hand, OL‐specific Sox10‐cre‐driven deletion of both Sox9 and Sox10 in mice leads to a substantial reduction of OPCs in the developing embryonic spinal cord, suggesting a compensatory mechanism between the two Sox family transcription factors (Finzsch et al., 2008). Importantly, Sox10 promotes the expression of PDGFRα, the receptor for the PDGF ligand, which regulates multiple aspects of OPC development (Calver et al., 1998). PDGF regulates OPC migration along with growth factors such as FGF‐2 and chemokines such as CXCL1 and CXCL12 (Dziembowska et al., 2005; Frost et al., 2009; Robinson et al., 1998; Vora et al., 2011). PDGF also acts as a mitogen, promoting survival, proliferation, and the maintenance of the OPC state in combination with growth factors FGF‐2 and IGF‐1 (Bansal et al., 1996; Goddard et al., 2001; Popken et al., 2004; Richardson et al., 1988) as well as neurotrophic factors BDNF and NT‐3 (Barres et al., 1993, 1994).

### 3.2 Positive regulators of OPC differentiation into OLs

Unlike in SCs where differentiation is reversible and plastic, OL differentiation in vivo seems to be a terminal event, necessitating tight control mechanisms to regulate myelination and maintain a large pool of OPCs throughout life. Much of OL development is thought to follow a general “de‐repression” model: OL differentiation proceeds intrinsically by default, and several inhibitory cues play important roles in regulating the timing and extent of differentiation at the transcriptional, epigenetic, and post‐translational levels (Egawa et al., 2019; Emery, 2010; Emery & Lu, 2015). In line with the de‐repression model, many of these transcription factors are also present in OPCs (He et al., 2007; Qi et al., 2001; Stolt et al., 2002; Sugimori et al., 2008; Weng et al., 2012; Xin et al., 2005), suggesting that inhibitory gene regulation mechanisms as well as extrinsic inhibitory factors modulate the activation of differentiation pathways at appropriate times.

Several transcription factors are known to promote OL differentiation, including Ascl1, Myrf, Nkx2.2, Olig2, Sox10, YY1, and Zfhx1b (Emery & Lu, 2015). Indeed, OL differentiation has been found to stall in the pre‐myelinating stage upon deletion of each of these factors in animal models, suggesting that they might work through parallel pathways to independently drive myelination. Olig2 is necessary to generate myelin and for the expression of mature OL markers including CC1 and myelin proteins in mice and zebrafish (Liu et al., 2007; Mei et al., 2013; Park et al., 2002; Zannino & Appel, 2009; Zhou et al., 2001). Its function in promoting OL differentiation may depend on its cooperation with the chromatin remodeler Brg1, which has been found to act on OL‐specific enhancers in primary rodent OPCs and in the mouse CNS (Yu et al., 2013). Sox10 has also been found to be crucial for maturation, working with the transcription factor Myrf to regulate a gene network promoting differentiation (Hornig et al., 2013; Stolt et al., 2002). Histone deacetylases (HDACs) and DNA methyltransferases (DNMTs) have also been found to strongly regulate OL differentiation, playing complex roles in both promoting and inhibiting differentiation in a stage‐specific manner (Egawa et al., 2019; Shen et al., 2005). Aside from their function in modifying histones, HDACs have been found to act in other ways to promote differentiation: HDAC1 works with YY1 to repress the transcription of inhibitory factors Id2, Id4, and Hes5 in primary mouse OLs (He et al., 2007; F. Ye et al., 2009). In addition, HDACs also prevent transcription of Wnt and Notch signaling target genes through inhibition and competition with downstream effector proteins, as demonstrated in developing chick and mouse spinal cords (Fancy et al., 2009; Kadam & Emerson, 2003; Sekiya & Zaret, 2007; F. Ye et al., 2009).

Myrf is a transcription factor thought to be a master regulator of myelin gene expression and is required for the initiation of myelination and its maintenance, as first demonstrated in vitro and in the developing chick spinal cord (Emery et al., 2009). In later studies in mice, Myrf has been found to be strongly induced during the early stages of differentiation and is not expressed strongly in OPCs. It directly targets a wide range of genes implicated in differentiation, including transcription factors, cytoskeletal and lipid metabolism genes, as well as myelin protein genes (Bujalka et al., 2013; Emery & Lu, 2015; Hornig et al., 2013; Koenning et al., 2012). Sox10 and Olig2 are known to bind to the enhancer regions of Myrf and are thought to induce its expression in cooperation with other proteins. In addition, there is significant overlap between Olig2, Myrf, and Sox10 binding sites, suggesting that myelin gene expression is regulated by all three proteins in mice (Bujalka et al., 2013; Hornig et al., 2013; Yu et al., 2013). While Myrf plays a central role in initiating myelination in OLs, it is not implicated in PNS myelination, which relies instead on the zinc finger transcription factor Krox20 (Britsch et al., 2001; Emery, 2013).

A variety of external signals have also been found to play important roles in regulating OL differentiation. Thyroid hormone and the insulin‐like growth factor IGF‐1 are both crucial for promoting morphological differentiation and the expression of myelin components such as MBP (Baas et al., 1997; Gao et al., 1998; P. Ye et al., 1995). Neurotrophic factors such as CNTF, NT‐3, and BDNF have also been found to promote expression of myelin genes, regulate sheath thickness and number, and promote survival of mature OLs in mice through a variety of pathways (Mayer et al., 1994; Rubio et al., 2004; Stankoff et al., 2002; Vondran et al., 2010; Xiao et al., 2010). On the other hand, while the NRG1/ErbB signaling axis is central for PNS myelination by SCs, it has not been found to play an equally important role in OLs. The timing of development, ultrastructure, and morphology were largely unaffected by the loss of NRG1‐Type III in mice during development (Brinkmann et al., 2008). Instead, NRG1 signaling may play larger roles in OPC survival and myelin plasticity and repair (Bartus et al., 2019; Colognato et al., 2002; Gauthier et al., 2013; Makinodan et al., 2012).

Formation of the compact myelin sheath involves high levels of cytoskeletal remodeling in OLs. As OLs reach the terminal stages of differentiation, they undergo disassembly of filamentous actin to enable lateral extension and myelin compaction (Nawaz et al., 2015; Zuchero et al., 2015). Actin‐severing proteins such as Gelsolin work in conjunction with actin assembly components such as Arp2/3 and formin proteins to form multiple layers of myelin through migration of the myelin inner tongue (Cristobal et al., 2022; Kim et al., 2006; Zuchero et al., 2015). Myelin sheath components MBP and CNP and small GTPases then facilitate cytoplasmic export to form compact myelin structures while maintaining cytoplasmic channels (Simons & Nave, 2016; Snaidero et al., 2017). Multiple upstream pathways and components have been implicated in regulating these complex mechanisms, including E3 ubiquitin ligases and the Wnt and Notch signaling pathways (Cristobal et al., 2022; López‐Juárez et al., 2017; Titus et al., 2017).

### 3.3 Negative regulators of OL differentiation

The major inhibitory factors that regulate OL differentiation include BMP, Notch, and Wnt signaling. BMP signaling induces the expression of the transcription factors Id2 and Id4, which can physically associate with Olig1/2 in primary mouse cells and may inhibit their activity (Samanta & Kessler, 2004). The Notch ligands Delta and Notch have been found to strongly inhibit OPC differentiation in vitro as well, acting through its downstream mediator Hes5 (Wang et al., 1998). Hes5 then binds to and competes with Sox10 in immortalized OL cells, preventing its activity at the MBP promoter and potentially blocking its binding at other sites as well. Indeed, Hes5 is known to play a net repressive effect on myelination as OL development begins to peak in postnatal mice (Liu et al., 2006).

**FIGURE 3:**
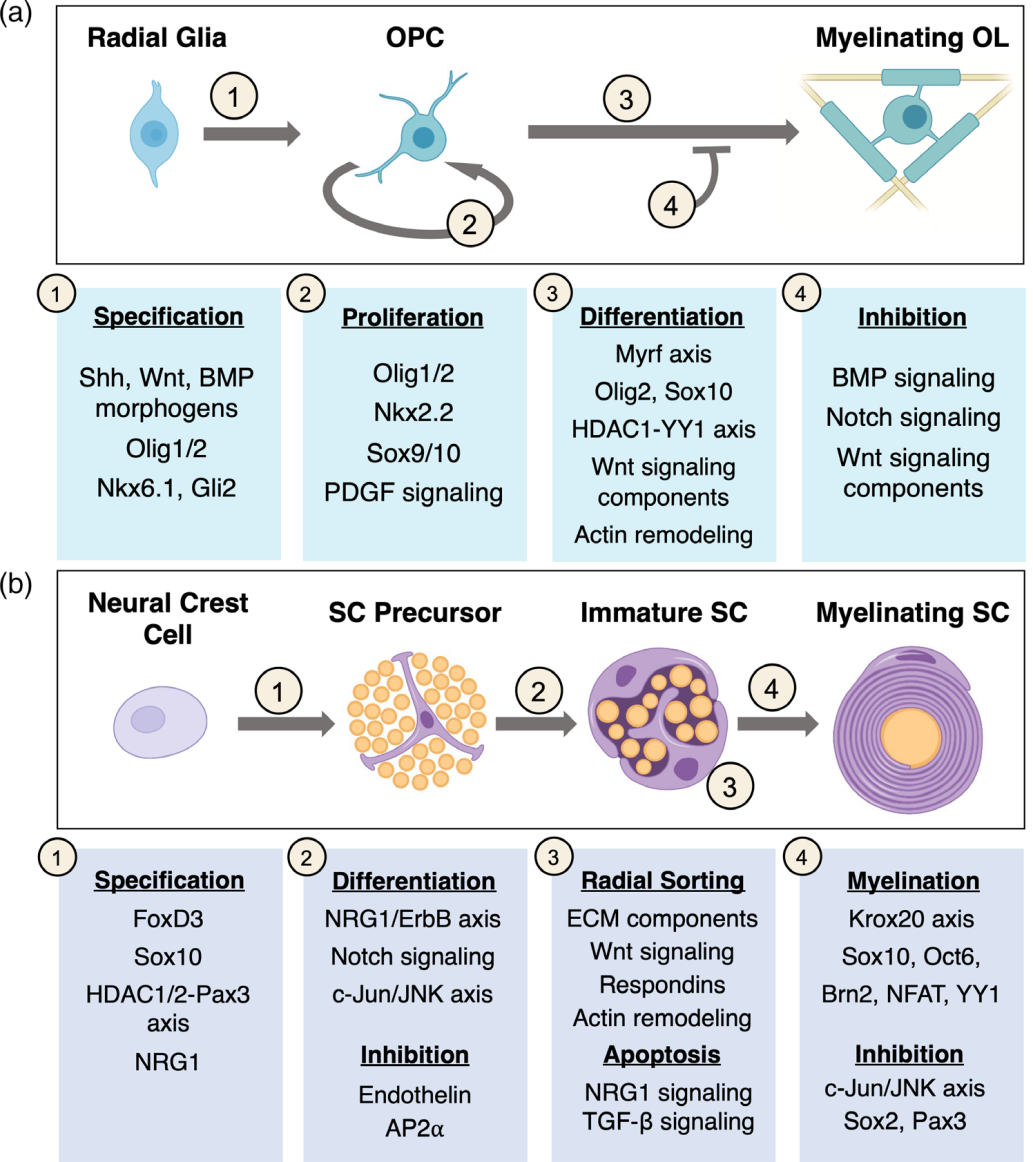
Molecular regulators of oligodendrocytes (OL) and Schwann cell (SC) development. (a) (1) Morphogen signaling plays a major role in the specification of radial glia into OPCs, activating transcription factors that define the OL lineage. (2) OPC proliferation is required to colonize the CNS and to maintain a stable OPC population. This process is dependent on both intrinsic transcription factors and signaling cascades downstream of the PDGF receptor. (3–4) OL development is thought to follow a de‐repression model, with intrinsic factors driving differentiation largely by default. Inhibitory extracellular signaling regulates the timing and extent of OL differentiation. The Wnt pathway plays a complex role, with different components promoting and inhibiting differentiation. (b) (1) Several molecules necessary for SCP specification are also expressed in crest cells. The factors that are sufficient to drive specification remain largely unknown. (2) Axon‐derived signals and positive and negative growth factors regulate SCP differentiation and survival. (3) External cues as well as cell number regulation are required to facilitate the complex process of radial sorting. (4) Krox20 is a master regulator of myelination and cooperates with other TFs to drive myelin gene expression. Inhibitory signals throughout differentiation play a large role in maintaining SC lineage plasticity.

Wnt signaling is known to be an important inhibitory pathway in OL differentiation but has complex stage‐specific roles in OL development. Wnt signaling components Tcf7l2 and β‐catenin form a complex that inhibits differentiation, but each one has been found to be necessary for full differentiation as well (Fancy et al., 2009; Fu et al., 2009). However, Tcf7l2 is transiently expressed during pre‐myelinating stages and can interact with HDAC1/2 to repress inhibitors of differentiation in mice (Ye et al., 2009); similarly, the deletion of β‐catenin has also been found to be detrimental to OL differentiation in the embryonic mouse spinal cord (Dai et al., 2014). In addition, intracellular signalosome components such as Daam2 and its downstream effectors have been found to mediate the activity of Wnt signaling during OL development and regeneration in mice (Cristobal et al., 2022; Ding et al., 2020; Lee et al., 2015). Thus, Wnt signaling is thought to promote some aspects of OL differentiation, but downregulation of Wnt signaling at intermediate stages may be necessary for OLs to reach their full differentiation state. An overview of the relevant mechanisms regulating each step are outlined in Figure 3a.

## 4 SIGNALING PATHWAYS AND TRANSCRIPTIONAL MECHANISMS REGULATING PNS MYELINATION

### 4.1 Specification and commitment from neural crest cells to SCPs

The signals that control the emergence of SCPs remain poorly studied due in part to the lack of markers that can clearly define the entire neural crest population (Jessen et al., 2015). Indeed, the transcription factors that are sufficient to specify glial differentiation from the crest have not yet been identified and many of the known molecules necessary for SC specification have significant overlap with crest cells. FoxD3 is a transcription factor that promotes the development of NCCs from early neural progenitors in the neural tube and is also expressed at early stages of the SC lineage. Functional experiments in the chick and mice showed that FoxD3 regulates the balance between neuronal, melanocyte, and glial fates and plays crucial roles in determining SC fates (Adameyko et al., 2012; Jessen et al., 2015; Nitzan et al., 2013). Sox10 is another transcription factor expressed in crest cells and known to be important in generating SC precursors. While Sox10 does not directly promote gliogenesis, it has been found to directly regulate important signaling processes in SCP development including NRG1 response and the expression of the FABP7 among others in mice (Britsch et al., 2001; Jacob et al., 2014). HDAC1 and HDAC2 are epigenetic regulators that cooperate with other proteins to initiate the formation of SCPs from crest cells. Previous studies in mice have found that HDAC1/2 cooperate with Sox10 to activate the expression of Pax3; subsequently, Pax3, Sox10, and HDAC1/2 function as part of a complex that promotes expression of glial lineage markers like P0 from NCCs (Jacob et al., 2014; Wahlbuhl et al., 2012).

NRG1 type III is a well‐studied molecule that binds to ErbB receptors in SC lineage cells and affects differentiation and proliferation at different stages of development (Leimeroth et al., 2002; Lyons et al., 2005; Shah et al., 1994). NRG1 type III is an axon‐derived factor that is required for the survival of SCPs. This factor is not essential for gliogenesis but has been found to suppress differentiation into neuronal cell fates (Jessen et al., 2015). Thus, while it is not directly responsible for specification, it is thought to play a major role in suppressing other differentiation options for crest cells and stimulates SC division once committed. Pathways downstream of NRG1/ErbB3 have been found to lead to the production of key SC markers through the activity of transcription factors like Sox10, Sox2, NF‐KB, Oct6, and Brn2 (reviewed in Salzer, 2015). Another important complementary pathway for specification and subsequent differentiation is Notch signaling. Notch signaling elevates the expression of ErbB3 receptors in SCPs, increasing the effectiveness of NRG1 signaling in embryonic mouse peripheral nerves and primary SCs (Woodhoo et al., 2009). While it is also unlikely to be involved in direct gliogenesis from crest cells, inactivation of this pathway delays subsequent differentiation from SCPs to SC lineage cells (Jessen et al., 2015; Woodhoo et al., 2009).

After specification into SCPs, these cells remain multipotent and can give rise to non‐SC fates. The NRG1 and Notch pathways are known to suppress the generation of fibroblast‐like cells and contribute to SC differentiation (reviewed in Jessen & Mirsky, 2005), and recent evidence from zebrafish studies has shown that the Wnt/β‐catenin pathway governs the decision between melanocyte and SC fates for SCPs (Colombo et al., 2022). Axon‐derived signals such as NRG1, Notch ligands, and endothelins regulate the phenotypic changes associated with this transition, and both NRG1 and Notch signaling can stimulate maturation from SCPs into SCs (Leimeroth et al., 2002; Shah et al., 1994; Woodhoo et al., 2009). SCP differentiation into iSCs is also accompanied by a strong increase in proliferation. As mentioned earlier, survival of iSCs ceases to be axon‐dependent and instead relies on autocrine signaling for survival and proliferation (Jessen & Mirsky, 2005; Meier et al., 1999). The production of the survival factors IGF2, NT3, PDGFB, and LIF have been known to occur at this transition period (Cheng et al., 2000; Dowsing et al., 1999; Jessen & Mirsky, 2005; Meier et al., 1999; Mogha et al., 2013).

### 4.2 Pathways regulating radial sorting

Radial sorting is a complex process known to be driven by several pathways, but the exact mechanisms remain an active area of exploration. Several studies have demonstrated that much of this process is dependent on ECM components in the basal lamina including laminins, collagen, and their corresponding receptors (Berti et al., 2011; Feltri et al., 2002; Occhi et al., 2005; Patton et al., 2008; Pellegatta et al., 2013; Rasi et al., 2010; Saito et al., 2003; Wallquist et al., 2005). Signaling molecules from axons such as Wnt and Respondins may also play roles in signaling phenotypic changes in SCs that mediate radial sorting (Grigoryan et al., 2013; Lewallen et al., 2011). In line with the complex spatial changes in nerve structure because of radial sorting, several downstream pathways that regulate the actin cytoskeleton have been identified to be crucial for this process, including FAK, Rho GTPases, Profilin, and N‐WASp (Benninger et al., 2007; Grove et al., 2007; Guo et al., 2012; Montani et al., 2014).

Precise control of SC number is required to facilitate the process of radial sorting and subsequently myelination. While iSCs do not rely on axons to survive, axon‐associated signals have been found to be important for their proliferation (Jessen & Mirsky, 2005; Salzer & Bunge, 1980). NRG1 from axons and laminins from the basal lamina are also known to promote SC proliferation in this process (Morrissey et al., 1995; Yu et al., 2005). Other pathways such as Notch, TGF‐B, and the Hippo pathways have also been implicated in driving SC proliferation during development (Atanasoski et al., 2004; Einheber et al., 1995; Feltri et al., 2016; Ridley et al., 1989). Death signaling is a complementary process that is important for the process of radial sorting and 1:1 matching of axons with myelinating SCs, as first described through comprehensive electron microscopy of postnatal rat nerves (Jessen & Mirsky, 2005; Webster et al., 1973). The only signaling pathway to date known to promote SC death during normal development is the TGF‐β signaling pathway. Interestingly, application of TGF‐β alone in cultured SCs induces apoptosis, but the addition of TGF‐β into media containing NRG1 induces DNA synthesis, implying a possible effect on proliferation (Parkinson et al., 2001; D'Antonio et al., 2006; reviewed in Woodhoo & Sommer, 2008). Together, these data suggest a cooperative effect between these pathways, where TGF‐β amplifies the proliferation of cells with a sufficient degree of axonal contact and thus NRG1 signaling activation. On the other hand, iSCs with weak axonal contact and thus low levels of NRG1 signaling are eliminated through the activation of apoptotic pathways.

### 4.3 Positive regulators of SCP differentiation into myelin SCs

After radial sorting, iSCs in contact with large‐caliber axons further differentiate to become pro‐myelinating and then myelin SCs. The transition to myelin SCs is known to be controlled in large part by Krox20, a master regulator of PNS myelination. Krox20 is necessary for myelin formation and maintenance, driving transcription of structural proteins and lipid synthesis genes needed for myelin formation, and Krox20‐null mice experience severe peripheral myelination deficits (Le et al., 2005; Topilko et al., 1994). Several upstream factors have been found to promote Krox20 upregulation, including Sox10, Oct6, Brn2, NFATc4, and YY1 (He et al., 2010; Jaegle et al., 2003; Kao et al., 2009; Weider et al., 2012); however, the external cues that activate these factors remains an open question. The maintenance of the myelinating phenotype in SCs requires the continuous expression of both Krox20 and Sox10, and adult deletion of either one in inducible SC‐specific cre mouse lines results in reversal of the differentiated state (Bremer et al., 2011; Decker et al., 2006). Importantly, the G protein‐coupled receptor Gpr126 also plays a crucial role in driving the differentiation program in the SC lineage. In mouse and zebrafish studies, Gpr126 was found to elevate the levels of cAMP and initiate numerous processes necessary for radial sorting, differentiation into myelinating and non‐myelinating fates, and overall peripheral nerve organization during development (Mogha et al., 2013, 2016; Monk et al., 2009, 2011). While evidence shows that Gpr126 is dispensable for maintenance of peripheral myelin, it plays a crucial role in the remyelination response (Fernandez et al., 2017; Mogha et al., 2016), indicating a potential repurposing of developmental pathways during regeneration.

Much like the CNS, PNS myelination has also been known to involve high levels of actin remodeling, albeit using different cellular components. For instance, during myelination, actin polymerization for process extension involves WAVE1 in OLs, with WAVE2 playing a parallel role in SCs (Bacon et al., 2007). Similarly, the roles of small GTPases Cdc42 and Rac1 and downstream pathways such as mTOR also play different roles in regulating myelin thickness, lamellipodia formation, and internode length (Benninger et al., 2007; Domènech‐Estévez et al., 2016; Jin et al., 2011). As in OL wrapping, actin depolymerization in SCs also plays a central role in the formation of compact myelin, and actin‐severing proteins Cofilin and Gelsolin have also been found to be active in SCs (Sparrow et al., 2012; Tanaka & Sobue, 1994). However, the upstream factors modulating their action differ compared to OLs (reviewed in Brown & Macklin, 2020). These demonstrate that myelin formation proceeds through distinct pathways and components between OLs and SCs, and further studies will be necessary to identify the factors responsible for differences in sheath number and myelin composition between these cell types. The mechanisms promoting differentiation into non‐myelinating SCs are outside of the scope of this review and are covered in detail in Harty and Monk (2017).

### 4.4 Negative regulators of myelin SC differentiation

As earlier noted, SCPs retain the ability to differentiate into non‐SC fates such as fibroblasts and melanocytes. Several factors have been found to inhibit differentiation, regulating the timing of later processes and retaining the multipotent pool of SCPs during development. Endothelin and the endothelin B receptor promote the survival of SCPs but inhibit maturation in embryonic rat nerves and cultured SCPs (Brennan et al., 2000). The only transcription factor known so far to have a role in this transition is AP2α. AP2α delays SC generation from SCPs when overexpressed in primary SCPs and is known to be downregulated when SCPs differentiate into iSCs (Stewart et al., 2001). Once committed to the SC fate, pathways that inhibit SC myelination play key roles in regulating the timing and extent of myelination. The c‐Jun/JNK pathway is required for NRG1 and TGF‐β signaling at earlier stages of SC development but is inhibitory to myelination and is inactivated at this stage, as demonstrated in vitro and in early postnatal rat nerves (Parkinson et al., 2004). Similarly, Notch signaling as well as the transcription factors Pax3 and Sox2 play important roles earlier in SC development but become negative regulators during the myelination stage in mice (Kioussi et al., 1995; Le et al., 2005). Both Sox2 and c‐Jun have been demonstrated to inhibit the expression of the myelin master regulator Krox20 in mice, which allows SCs to adopt alternate cell fates (Parkinson et al., 2008; Roberts et al., 2017).

c‐Jun in particular plays a distinct role as a master regulator of the repair SC phenotype. This transcription factor is upregulated in iSCs but remains relatively low in all other stages of myelinating and non‐myelinating SC development (Jessen & Mirsky, 2016; Parkinson et al., 2004, 2008). c‐Jun was found to be dispensable for development and maintenance in mice, but essential for many aspects of nerve repair by SCs (Arthur‐Farraj et al., 2012; Fontana et al., 2012; Huang et al., 2019). Evidence has suggested a wide‐ranging role for c‐Jun in regulating the transition to repair SCs through epigenetic, transcriptional, and post‐translational mechanisms (Arthur‐Farraj et al., 2012, 2017; Jessen & Mirsky, 2022), but the mechanisms governing all aspects PNS repair and remyelination are outside of the scope of this review and are covered in detail in Jessen and Mirsky (2016, 2019b). An overview of the relevant mechanisms regulating the formation of myelinating and non‐myelinating SCs is outlined in Figure 3b.

## 5 RECENT ADVANCES IN MYELIN PHYSIOLOGY

**FIGURE 4:**
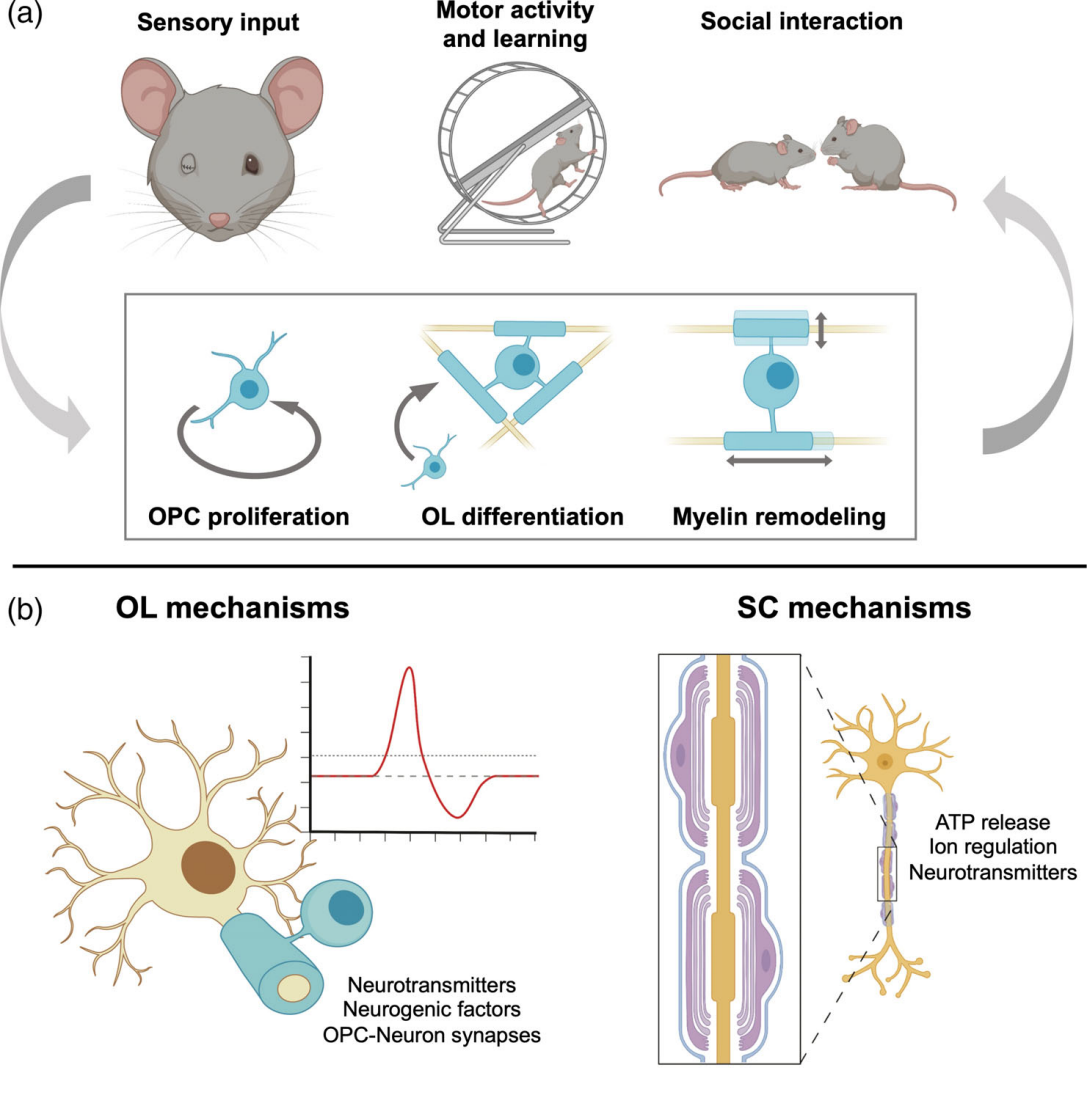
Recent advances in oligodendrocytes (OL) and Schwann cell (SC) development. (a) Advanced techniques such as longitudinal two‐photon imaging and serial electron microscopy have directly implicated experience‐modifying paradigms in modulating key aspects of myelin formation and remodeling. Emerging evidence has also increasingly revealed the crucial role of OL lineage cells in higher central nervous system (CNS) function. (b) Continuous communication between myelinating cells and neurons occurs in vivo: Neuronal activity induces changes in myelin and vice versa. While these have been demonstrated in both the CNS and PNS, recent evidence suggests distinct pathways in each.

The advent of new tools for manipulating and observing both neuronal and myelinating cell activity has contributed to a greater understanding of myelin's roles in CNS development and function. Here, we discuss major advances in three major areas of CNS and PNS development: (1) myelination in response to neuronal activity; (2) experience‐dependent myelin plasticity; and (3) myelinating glia modulating neuronal activity (see Figure 4).

### 5.1 Myelination in response to neuronal activity

Activity‐dependent mechanisms showing communication between axons and myelinating cells were first demonstrated through co‐culture experiments using mouse DRG neurons and OL‐ and SC‐lineage cells. Action potentials in mouse DRG neurons induced adenosine receptor activation in OPCs, subsequently inhibiting proliferation and promoting myelination by activating synthesis of myelin proteins (Stevens et al., 2002; Wake et al., 2011). Building on these discoveries, several important questions underlying activity‐dependent myelination were addressed using live imaging studies in zebrafish. Both synaptic and non‐synaptic vesicles were found to regulate the extent of myelination as well as the selection of axons to be myelinated (Hines et al., 2015; Mensch et al., 2015; Wake et al., 2015). One recent study also demonstrated that subsets of OPCs responded differently to neuronal activity and played different roles in the CNS: one subset playing an integrative role in signaling, with the other responsible for forming new myelin (Marisca et al., 2020). Further studies are necessary to know if these cellular dynamics are conserved in mammals as well.

Studies using neuronal stimulation in mice clearly showed that adaptive myelination also occurs the mammalian brain. Optogenetic activation of neurons in the premotor cortex led to thicker myelin in associated white matter tracts (Gibson et al., 2014), showing the first direct link between neuronal activation and myelin structure in vivo. Later studies demonstrated that stimulated axons had a higher probability of being myelinated and had thicker sheaths (Mitew et al., 2018). Mechanistically, recent work has found that morphological cues (Stedehouder et al., 2018), nutritional availability (Kohnke et al., 2021), BDNF release (Geraghty et al., 2019), and neurotransmitter release (Benamer et al., 2020; Etxeberria et al., 2016) resulting from neuronal activity may be key signals in regulating OPC proliferation and differentiation.

In the PNS, the differentiation state of SCs is known to be highly plastic and responsive to external cues, as evidenced by the robust injury response in peripheral nerves. As such, it remains difficult to disentangle SC differentiation from the variety of external cues including immune cells and the basal lamina. Early work using cultured mouse DRGs and SCs showed that electrical stimulation and ATP release from neurons initiated intracellular Ca^2+^ waves in SCs in vitro (Stevens et al., 1998; Stevens & Fields, 2000), implicating purinergic receptors as key mediators of neuron‐to‐SC signaling (Stevens & Fields, 2000). Pathways downstream of ATP receptors have also been identified in recent years, including mitochondrial Ca^2+^ to promote myelination in early postnatal rat nerves (Ino et al., 2015) and GPCR signaling to suppress SC growth in adult mouse nerves (Coover et al., 2018). In addition, modulation of SC myelination in response to neuronal activity has also been found to occur through GABA (Magnaghi et al., 2004), Na^+^, K^+^, and Cl^−^ signaling (Heredia et al., 2018; Marshall‐Phelps et al., 2020) and glutamate release (Hu et al., 2019).

### 5.2 Experience‐dependent myelin plasticity

Experiential paradigms better recapitulate the complex, multicellular response to external inputs to the nervous system. Studies by Makinodan et al. (2012) and Liu et al. (2012) first showed persistent impairments in myelination in the prefrontal cortex as a result of social isolation in mice. In recent years, new tools such as longitudinal two‐photon microscopy and newly generated OL‐specific reporter mouse strains have allowed detailed investigation of myelin dynamics in the CNS. Modifying sensory inputs through monocular deprivation (Etxeberria et al., 2016; Osanai et al., 2018; Yang et al., 2020) and whisker removal (Hughes et al., 2018; Osanai et al., 2018) were found to decrease oligodendrogenesis, de novo myelin formation, sheath length, and nerve conduction velocity in the brain and optic nerve in mice. Conversely, sensory enrichment induced a robust increase in the integration of new OLs and myelin without changing the length of existing sheaths (Hughes et al., 2018). Other behavioral paradigms such as motor learning (Bacmeister, 2020) and forced swim stress (Osso et al., 2021) induced similar effects including enhanced OPC differentiation and greater myelin remodeling in mice.

Mechanistically, NRG1 and its receptor ErbB3 have been implicated in the OL response to social isolation in adolescent mice (Makinodan et al., 2012). In monocular deprivation studies in P7‐P32 mice, ablation of glutamatergic signaling from neurons phenocopied the shortening of myelin sheaths in the retina (Etxeberria et al., 2016) suggesting a role for glutamate in myelin plasticity. On the other hand, monocular deprivation induces myelin sheath remodeling only in GABAergic neurons in the adult mouse visual cortex, suggesting a yet unidentified mechanism unique to inhibitory neurons (Yang et al., 2020). Other pathways such as dynorphin and the kappa opioid receptor (Osso et al., 2021), Wnt signaling (Zheng et al., 2019), and endothelin (Swire et al., 2019) may also play roles in activity‐dependent myelin plasticity. While these shed light on the complexity of the myelin plasticity response, it is still unknown whether the pathways are generalizable across the CNS or if they are circuit‐ and location‐specific. In addition, the relative contribution of different cell types to myelin plasticity remains an active area of exploration.

While most research on experience‐dependent myelination in the PNS has been confined to resistance and aerobic exercise paradigms, recent evidence has shown parallel results as studies in the CNS, where increasing external stimuli tend to promote myelination. In rats, load‐carrying tasks and ladder‐based resistance training increased myelin sheath thickness in the tibial nerve (Krause Neto et al., 2017) and the radial and sciatic nerves (Neto et al., 2021). The in vivo mechanisms underlying this response are still largely unknown, but one study has identified that aerobic exercise may induce increases in peripheral myelin thickness in a BDNF‐ and VEGF‐dependent manner (Sakita et al., 2018), suggesting contributions of multiple cell types in the PNS.

### 5.3 Myelinating glia modulating neuronal activity

Recent progress in the field has clearly demonstrated a reciprocal relationship between neurons and OLs. Seminal studies clearly showed that oligodendrogenesis and differentiation are necessary for higher behavioral functions in mice, including social interaction, learning and memory, as well as motor learning tasks (Makinodan et al., 2012; McKenzie et al., 2014; Xiao et al., 2016). Since then, other studies have expanded on these concepts, finding a similar necessity for OL function in spatial memory (Steadman et al., 2020; Wang et al., 2020), fear memory (Pan et al., 2020), and sensory discrimination (Benamer et al., 2020).

The interplay between neurons and OLs and mechanisms by which OLs modulate neuronal function are only beginning to be explored. Myelination may regulate neuronal activity through modulation of conduction velocity (Kato et al., 2020; Micheva et al., 2021) and regulation of extracellular ion concentrations (Larson et al., 2018). Benamer et al. (2020) demonstrated that OPC‐neuron GABAergic synapses play an important role in circuit formation, regulating firing frequency and conduction velocity in fast‐spiking interneurons during postnatal cortical development in mice. Indeed, OPC‐specific inactivation of the GABA_A_ receptor in mice led to a loss of post‐synaptic currents in OPCs and changes in myelin structure and OL morphology; however, the downstream mechanisms mediating these effects remain unknown. Studies in zebrafish have identified the role of OPCs in the formation and tuning of visual circuits in development (Xiao et al., 2022). Using serial EM and computational reconstruction paired with single‐cell RNA‐sequencing, recent unpublished work (Buchanan et al., 2021) has also demonstrated that OPCs phagocytose axon branches and presynaptic terminals in the visual cortex in mice. These studies reveal yet greater roles for OL lineage cells at different stages than previously thought.

SCs are known to play active roles in modulating neuronal function during PNS development due to their multipotency. Myelinating SCs in mice have been found to regulate conduction speed and through restricting axonal diameter (Eichel et al., 2020). Remak SCs are known to maintain smaller caliber axons in bundles, and defects in axonal sorting in mice have been found to result in neuropathic pain, possibly due to neuron hyperactivity (Orita et al., 2013). Non‐myelinating perisynaptic SCs are required for the formation and maintenance of neuromuscular junctions during development (Barik et al., 2016) and play an active role in the elimination and strengthening of synaptic terminals (Darabid et al., 2018). Recent work has also identified a specialized subpopulation of cutaneous SCs in mice that work as mechanosensory cells and are sufficient to initiate action potential propagation (Abdo et al., 2019). Together, these demonstrate the high level of SC plasticity that allows this cell lineage to play diverse roles in regulating peripheral neuron activity. Indeed, single‐cell RNA sequencing studies in zebrafish have only begun to uncover the diversity of peripheral glia, revealing distinct mechanisms that give rise to SCs in different regions of the PNS (Tasdemir‐Yilmaz et al., 2021). Together, these studies reveal that myelinating cells play crucial roles in the development of the nervous system and represent promising areas of exploration to better understand CNS and PNS function.

## 6 CONCLUDING REMARKS

On the surface, OLs and SCs seem to play similar roles as the sole producers of myelin in the nervous system. However, they comprise two highly distinct cell types that undergo unique developmental programs and utilize different strategies to maintain plasticity in their respective systems. The CNS maintains OL lineage cells at multiple stages of differentiation to serve different purposes: mature OLs to provide myelin, and OPCs to serve as a highly responsive pool of stem‐like cells that play a variety of roles. On the other hand, the PNS utilizes the plasticity of the neural crest‐derived SCs to produce both myelinating and non‐myelinating cell fates. Fully differentiated SCs depend on environmental cues to maintain their phenotypes and can radically change their phenotypes in response to injury.

Owing to their cell lineage and unique environments, the molecular machinery used over the course of OL and SC development diverge greatly. However, key aspects of their differentiation programs are similar, including the production of many common myelin components as well as mechanisms that allow them to respond to neuronal activity. Understanding both the shared and distinct characteristics of each cell lineage will be essential to identifying appropriate therapeutic strategies for both the CNS and PNS.

## Data Availability

Data sharing is not applicable to this article as no new data were created or analyzed in this study.
